# From Bulk to Single Molecules: Surface-Enhanced Raman Scattering of Cytochrome C Using Plasmonic DNA Origami Nanoantennas

**DOI:** 10.1021/acs.nanolett.4c00834

**Published:** 2024-06-03

**Authors:** Amr Mostafa, Yuya Kanehira, Kosti Tapio, Ilko Bald

**Affiliations:** Institute of Chemistry, University of Potsdam, Potsdam 14469, Germany

**Keywords:** DNA origami, SERS, Single molecules, Cytochrome C, Amide III

## Abstract

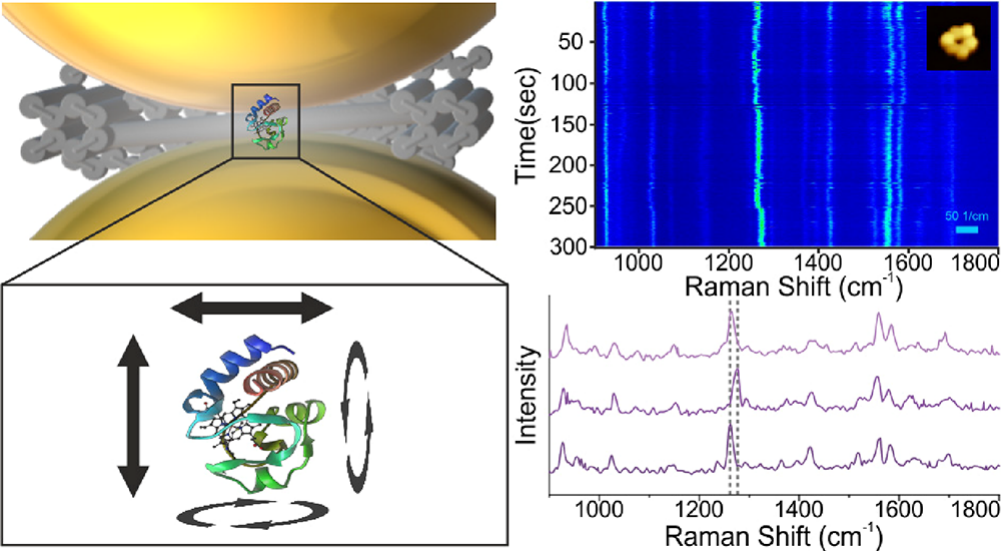

Cytochrome C, an evolutionarily conserved protein, plays pivotal roles in cellular respiration and apoptosis. Understanding its molecular intricacies is essential for both academic inquiry and potential biomedical applications. This study introduces an advanced single-molecule surface-enhanced Raman scattering (SM-SERS) system based on DNA origami nanoantennas (DONAs), optimized to provide unparalleled insights into protein structure and interactions. Our system effectively detects shifts in the Amide III band, thereby elucidating protein dynamics and conformational changes. Additionally, the system permits concurrent observations of oxidation processes and Amide bands, offering an integrated view of protein structural and chemical modifications. Notably, our approach diverges from traditional SM-SERS techniques by de-emphasizing resonance conditions for SERS excitation, aiming to mitigate challenges like peak oversaturation. Our findings underscore the capability of our DONAs to illuminate single-molecule behaviors, even within aggregate systems, providing clarity on molecular interactions and behaviors.

Cytochrome C (CytC) is a foundational protein found in the mitochondria of eukaryotic cells and in bacterial cytoplasm.^1^ Characterized by its single polypeptide chain of about 100 amino acids and a covalently attached heme prosthetic group^2^ (Figure 1A), it plays a critical role in oxidative phosphorylation. Evolutionarily, the sequence conservation of CytC across diverse organisms highlights its biological importance and has made it a benchmark for comparative genomics. Beyond its role in energy production, CytC is pivotal in apoptosis, a regulated cell death mechanism essential for maintaining cellular balance.^3^ Its involvement in these processes underscores its relevance in cellular biology and the importance of understanding its functions and dysregulations.

**Figure 1:**
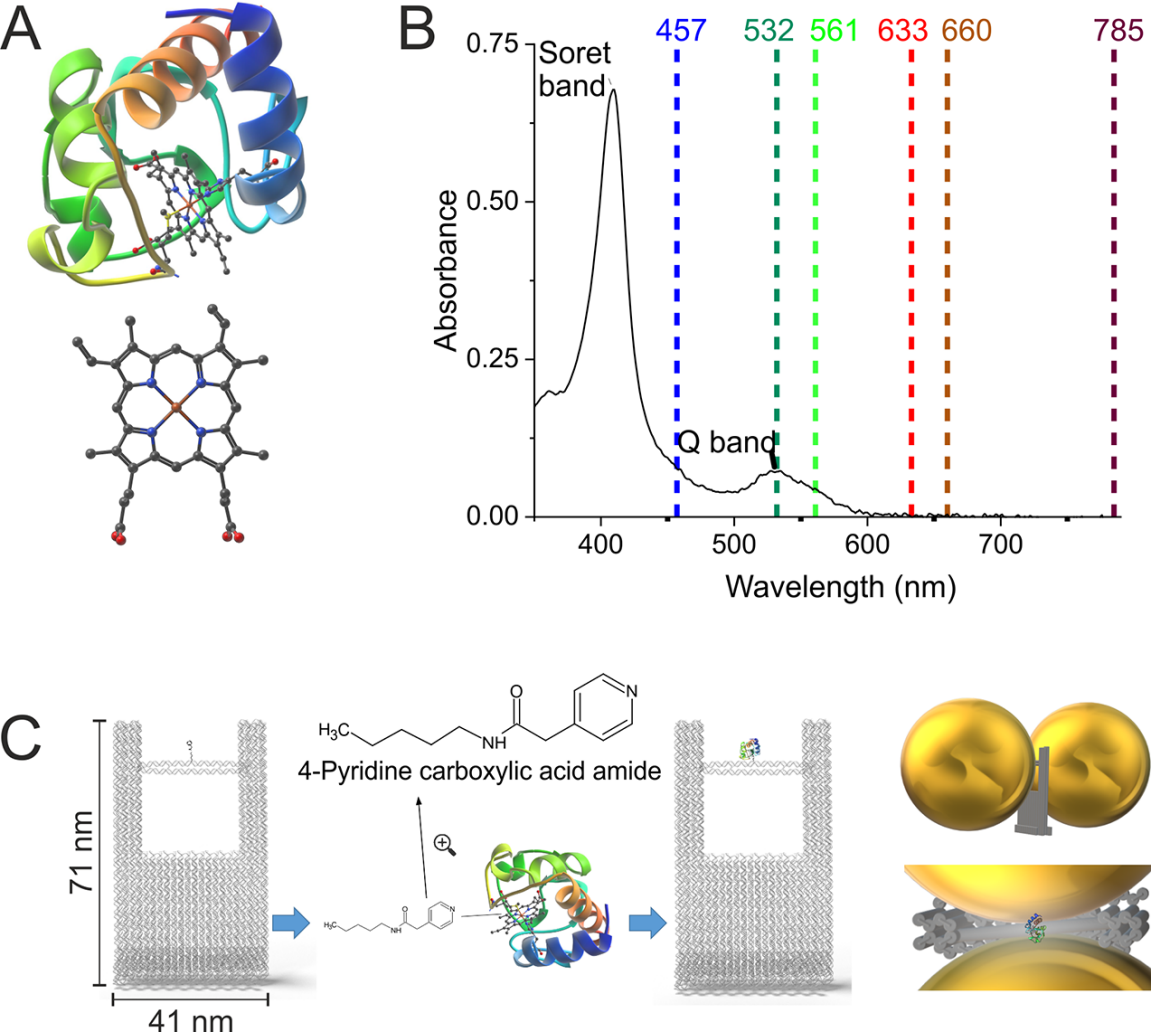
(A) Schematic model of CytC with the heme unit visible in the center. Underneath is a model of the heme unit. (B) UV–visible absorption spectrum of powder CytC. The vertical lines show the laser positions used in our study. (C) A schematic model of the DNA Nanofork, showing the attachment of CytC to the DNA Nanofork and the subsequent attachment of the AuNPs to form the DONA structure. The structure of CytC is based on the crystal structure 2b4z deposited at the protein data bank (PDB): https://www.rcsb.org/structure/2b4z.^29^

Raman spectroscopy has been a common tool for studying CytC, offering insights into the protein’s secondary structure and the state of its heme group.^4^ With technological advancements, surface-enhanced Raman scattering (SERS) has become increasingly utilized, providing detailed information even at very low concentrations of CytC.^5^

The recent advancements in single-molecule (SM) SERS have undeniably ushered in a new era of molecular study.^6^ This innovative approach promises unparalleled insights into molecular systems, especially proteins. Through the merger of SM-SERS with gold nanopores,^7^ the scientific community is presented with an opportunity to scrutinize individual protein segments in ways previously considered unattainable, offering the potential of SM protein sequencing.

From the pioneering demonstrations of SM-SERS by Nie and Emory in 1997,^8^ which showcased the potential of observing individual molecular behavior, contemporary research has navigated toward perfecting hotspot enhancement and crafting nanoscale devices tailored for superior signal consistency. Parallelly, efforts have been channeled into the development of novel nanostructures. Techniques such as nanosphere lithography^9^ and electron beam lithography^10^ have gained prominence, highlighting the community’s commitment to pushing the boundaries of the possible.

Nevertheless, despite the remarkable strides, the journey is fraught with challenges. Strategies like “shell-isolated nanoparticle-enhanced Raman spectroscopy” (SHINERS),^11^ though promising, come with their own set of limitations such as limited sensitivity^12^ and signal enhancement challenges.^13^ The intricate task of data interpretation in SM-SERS remains a daunting challenge.^14^

The complexity of proteins further compounds these challenges. Huang and his team illuminated the potential of SM-SERS in identifying specific amino acid residues in proteins, leveraging nanoparticles.^15^ The prospect of real-time monitoring of protein interactions through SERS tags has added another dimension to this research. By amalgamating SM-SERS with other established tools, like atomic force microscopy,^16^ there’s potential to demystify protein–ligand interactions and the enigmatic world of protein folding dynamics. However, the inconsistency of SERS substrates remains a consistent challenge.^17^ This highlights the pressing need for a universally adaptable method tailored for protein studies.

DNA origami provides a unique approach to studying molecules by SERS at a single molecule level.^18−22^ Our DONA system represents a significant development in DNA origami technology, particularly noted for its versatile applications.^23,24^ It is designed to assemble gold (Au) or silver (Ag) nanoparticle dimers with adjustable gap sizes, reaching as small as 1.17 nm. This feature notably enhances surface-enhanced Raman scattering (SERS) signals, enabling the detection of single-molecule SERS signals.^25^ Furthermore, the DONA system can accommodate molecules of varying sizes across different excitation wavelengths, showcasing its adaptability to a range of experimental needs. By correlation of SERS measurements and atomic force microscopy (AFM), we follow for the first time the SERS signals of a single protein over an extended time to leverage the full potential of SM-SERS. For this study, CytC is strategically attached to the DONA via a DNA staple modified with 4-pyridine carboxylic acid amide (Figure 1).^26^ This modified staple is centrally positioned within the bridge structure to ensure optimal placement of CytC within the SERS hotspot. We aim to provide a closer look at the peptide backbone signals, which could give a more detailed perspective on molecular behaviors at the single-molecule level. Historically, shifts in the Amide I and Amide III bands, resulting from peptide bonds within proteins, have been indicative of protein structural changes.^27^ Our system aims to offer clearer insights into CytC’s dynamics by focusing on these shifts and simultaneously observing oxidation states of the central iron ion, which can be concluded from signals around 1363 and 1373 cm^–1^ for the reduced (Fe^2+^) and oxidized state (Fe^3+^), respectively.^28^ Furthermore, our approach diverges from traditional resonance-focused methods in SM-SERS, offering a fresh methodology. This adjustment might allow for a more detailed observation of the molecular landscape, potentially minimizing peak overshadowing.

The Amide III bands are crucial in protein studies due to their sensitivity and specificity. In Raman and infrared spectra, the Amide III band of proteins typically manifests within a wavenumber range of 1200 to 1350 cm^–1^. This band results from the combined N–H bending and C–N stretching vibrations in the protein’s peptide backbone.^27^ The precise location of the band within this range can change based on the protein’s secondary structure. For example, alpha-helices often contribute to peaks in the 1265–1300 cm^–1^ range, beta-sheets are generally observed around 1229–1235 cm^–1^, and random coils tend to appear in the 1243–1253 cm^–1^ range.^30^

In the present work, we demonstrate the fabrication of DNA origami-based SERS substrates that allow the monitoring of the Amide III band in the green to red part of the optical spectrum, i.e. out of resonance up to the single-molecular level.

We will present first a basic characterization of the CytC, employing UV–vis absorption and normal Raman (NR) scattering. Subsequently, the focus shifts to SERS to probe deeper into the molecular details. This stepwise approach, from general to specific, enables us to observe and analyze molecular characteristics, extending our insights to the single-molecule (SM) level.

The utility of UV–visible absorption spectroscopy in the study of CytC primarily focused on distinguishing between the oxidation states of the iron ion. The ferric (Fe^3+^) state of the iron ion in CytC is accompanied by a distinctive Soret band in the visible and near-ultraviolet region, pinpointed specifically at a wavelength of 408 nm (Figure 1B). This band’s presence served as a clear marker for the ferric state. Alongside this, another spectral feature, the Q-band, which is commonly associated with electronic transitions in proteins that harbor a heme group, was discerned at a wavelength of 530 nm. For the ferrous iron (Fe^2+^) the Soret band is at 416 nm, the Q-band at 520 nm, and an additional spectral feature at 550 nm. Collectively, these spectral characteristics allowed for a confident inference that the iron ion in our CytC solution predominantly exists in the ferric (Fe^3+^) state.^31^

Our exploration using NR scattering was carried out on powdered CytC samples. This involved the systematic use of various laser wavelengths to discern their influence on spectral peak intensities and positions (Figure 2A). Among the lasers tested, the 532 nm laser stood out, delivering the most optimal signal-to-noise (S/N) ratio. However, a broader wavelength range, spanning from 561 to 633 nm, also provided satisfactory S/N ratios. At wavelengths of 457 and 785 nm, signals were broader, complicating the clear identification of distinct bands.

**Figure 2:**
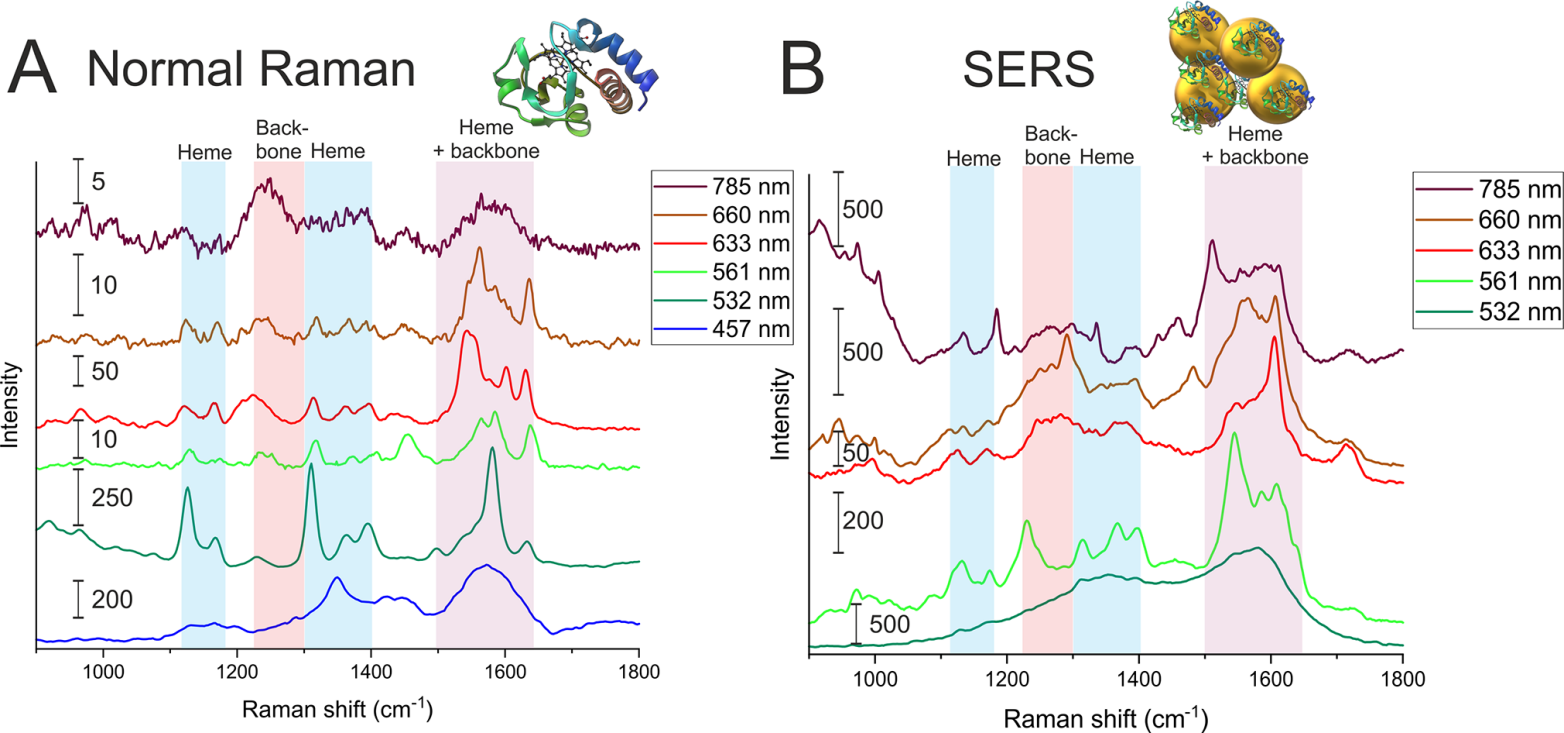
(A) Normal Raman (NR) spectra of powder CytC using different excitation lasers (blue zones highlight heme unit peaks, the red zone indicates peptide backbone peaks, and the purple zone shows spectral contributions from both). (B) SERS spectra of powder CytC mixed with 60 nm AuNPs using different lasers (blue zones highlight heme unit peaks, the red zone indicates peptide backbone peaks, and the purple zone shows spectral contributions from both). More detailed band assignments are summarized in Tables SI1 and SI2.

Intriguingly, we observed distinct spectral trends associated with different laser wavelengths. Lasers operating at 457 and 532 nm highlighted peaks characteristic of the heme unit, particularly in the 1120 to 1180 cm^–1^ and 1350 to 1400 cm^–1^ regions. Conversely, lasers at wavelengths of 633, 660, and 785 nm presented less intense heme peaks. At 785 nm, the S/N ratio is poor due to lower scattering efficiency at higher wavelengths. Yet, at these longer wavelengths, peaks within the 1180 to 1350 cm^–1^ range became more clearly visible, likely attributable to the peptide backbone of CytC. There was also a noticeable variation in the broader region from 1500 to 1640 cm^–1^, which contains contributions from both the heme unit and peptide backbone. Validating the robustness of our experimental approach, we found that the peaks identified using the 532 nm laser were in strong alignment with patterns documented in established literature^29^ (Table SI1).

Our SERS reference experiments were performed with CytC solutions mixed with 60 nm gold nanoparticles (AuNPs). Subjecting this mixture to varying laser wavelengths (Figure 2B; the same as used for NR experiments except for 457 nm, because AuNPs do not provide sufficient SERS signal enhancement at this wavelength), we discovered that the 561 nm laser produced the best S/N ratio. An extended range, between 561 and 660 nm, consistently emerged as beneficial for obtaining a satisfactory S/N ratio in this specific system.

Interestingly, despite its relatively lower S/N ratio, the 532 nm laser registered the highest signal intensity; however, signals were broader, complicating the clear identification of distinct bands. This outcome aligns with the inherent absorbance characteristics of CytC and the recognized enhancement region of AuNPs. Building on the patterns observed in the NR experiments, lasers operating at 633, 660, and 785 nm showcased less intense peaks from the heme unit in the SERS setting, specifically in the 1350 to 1400 cm^–1^ region. Conversely, the 660 nm laser exhibited intense peaks between 1250 and 1310 cm^–1^, likely associated with the peptide backbone of CytC. Our comparative efforts, especially utilizing the 532 and 561 nm lasers in the SERS framework, revealed consistency between our observed peaks and those chronicled in the literature (Table SI2). Across both the NR and SERS methodologies, we encountered recurrent challenges in determining the oxidation state of the iron ion.^32^ These challenges stemmed from the simultaneous presence of peaks representing both the oxidized and reduced states in certain spectra. This ambiguity was further intensified by minor spectral shifts, particularly evident in spectra obtained with the 561 nm laser. Layering onto these challenges, the inherent complexity of both bulk Raman and SERS spectra added additional dimensions of difficulty, especially when considering the influence of diverse CytC molecular configurations present in the samples.

Conventional SERS spectra have the disadvantage that a large number of possible molecular configurations on the nanoparticles are possible, and the appearance of spectra depends strongly on the specific interaction between molecules and nanoparticles. Transitioning to SM-SERS with DONAs addresses these limitations, offering a more controlled and consistent analysis platform.

Using the 660 nm laser, we acquired a series of SM spectra from individual DONAs (Figure 3). These signals were transient, predominantly lasting between one to three seconds but occasionally extending up to 30 seconds (Figure 3A). Such fleeting signals could be attributed to either the CytC molecule’s movement in and out of the hotspot^33^ or the formation of new hotspots from temperature-induced gold nanoparticle melting due to laser interactions.^34^

**Figure 3:**
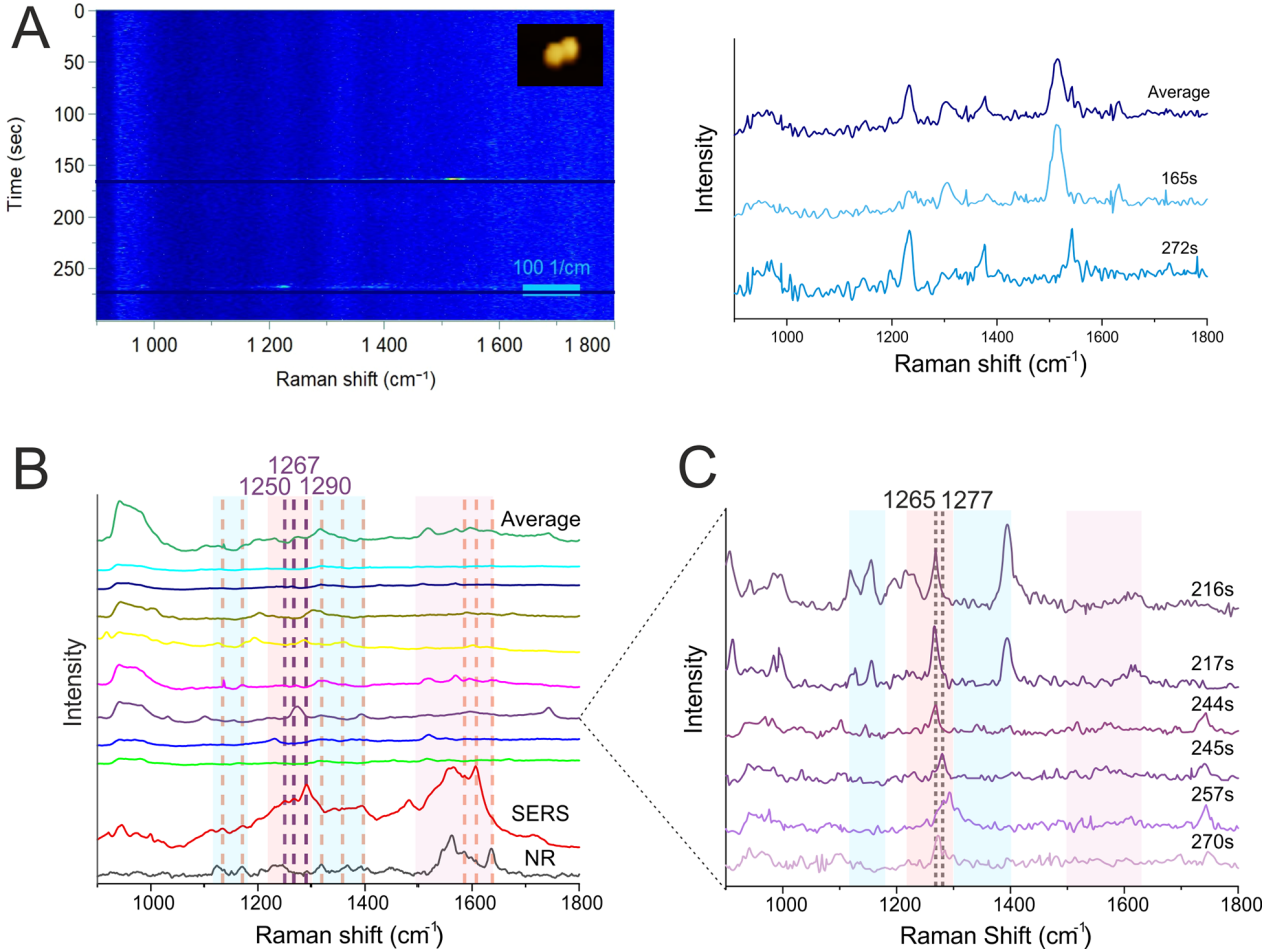
(A) Heat map of a single DONA measurement (left) with corresponding SERS signals at different time points (right). Inset: AFM image (325 nm × 240 nm, W × H) showing a single DONA with CytC. (B) SERS spectra showing measurements of individual DONAs averaged over time as well as the total average calculated from all single-DONA measurements, compared to reference NR and SERS of CytC (vertical orange lines represent bands observed in the NR and SERS references and literature, summarized in Table SI1 and Table SI2; purple lines represent bands observed in our SERS reference measurements and not assigned in the literature). Each spectrum represents the time average of a measurement from a single DONA (blue zones highlight heme unit peaks, the red zone indicates peptide backbone peaks, and the purple zone shows spectral contributions from both). (C) SERS spectra showing different time points from a single DONA measurement, highlighting the shift of the Amide III band (blue zones highlight heme unit peaks, the red zone indicates peptide backbone peaks, and the purple zone shows spectral contributions from both).

A key observation is the sharpness of the peaks, a hallmark of SM spectra. Upon accumulating the signals over the entire observation period, such time-averaged SERS spectra from individual DONAs (Figure 3B; the green spectrum labeled “average” represents a total average of all collected single-molecule SERS spectra) displayed recognizable CytC fingerprint peaks, shown as orange vertical lines in Figure 3B.^35^ However, no single spectrum captured all these peaks, suggesting variability in the enhancement of different vibrational bands due to the specific orientation of the CytC molecule within the hotspot.

Figure 3C presents individual SERS spectra of a single DONA at selected time points. The SM-SERS spectra are subject to transient changes in band position and intensity. The most prominent changes are associated with the Amide III peak. Since the Amide III band is composed of different contributions the apparent spectral shifts are likely due to a change of these different contributions, i.e., α helix and random coil structures, which might also change with the relative orientation of the protein within the SERS hotspot. Additionally, we observed that bands associated with the heme unit change intensity. Specifically, we observed a temporary spectral shift in the Amide III band from approximately 1265 cm^–1^ to 1277 cm^–1^. This shift lasted around 5 s before returning to its original position. Intriguingly, this shift coincides with a decrease in heme peak intensities. The disappearance of heme peaks just before the Amide III shift around the 244 s could suggest a reorientation of the CytC molecule within the hotspot. Subsequently, the appearance of both Amide III peaks around 257 s might indicate a mixed conformational state (Figure 3C).

A collective examination of the data revealed a comprehensive set of CytC fingerprint peaks, closely aligned with the 660 nm reference SERS spectrum. Interestingly, there were peaks in our study that were reported but not assigned in prior literature (Figure 3B, purple vertical lines). We assign these peaks between 1230 and 1300 cm^–1^ to the Amide III band.^36^

Transitioning from studying single DONAs to DONA aggregates it is observed that while SM-SERS spectra exhibit strong fluctuations, DONA aggregates provide more stable conditions for CytC analysis and yield higher signal intensities. We collected spectra from small aggregates composed of dimer clusters, typically encompassing 6–12 nanoparticles (Figure 4). These aggregates likely form either through the drying effect or reduced electrostatic repulsion between AuNPs due to ionic screening.^37^ In stark contrast to the dimer observations, emission signals from these aggregates were significantly more persistent, often spanning the full 5 min measurement duration (Figure 4A). Although up to six CytC molecules could be present in such an aggregate, it needs to be noted that not all DNA origami structures carry a CytC molecule due to a limited affinity of CytC to pyridine. Hence, this extended signal duration is attributed to a larger collective hotspot area and better stabilization of CytC within a hotspot.

**Figure 4:**
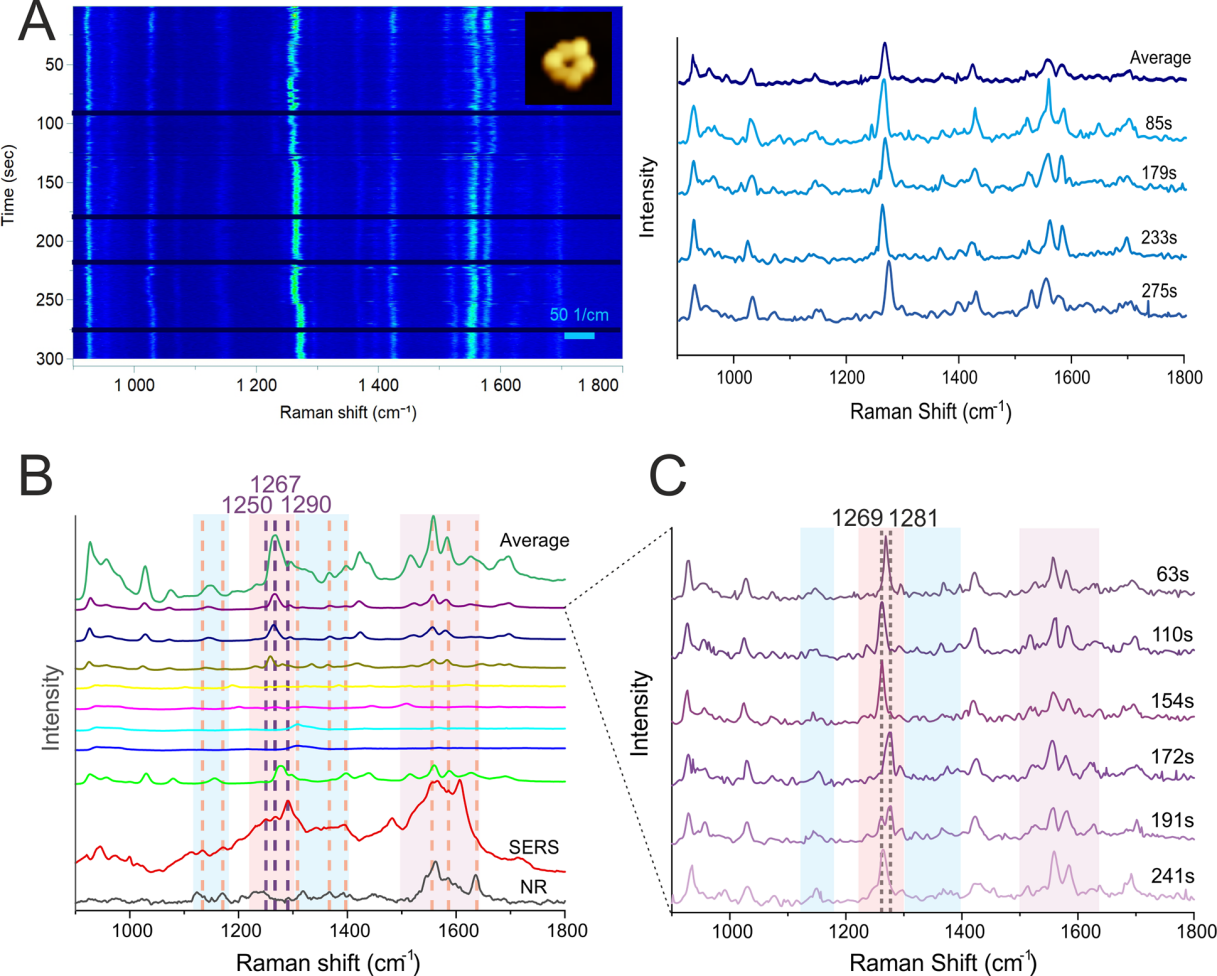
(A) Heat map of a DONA aggregate measurement (left) with corresponding SERS signals at different time points (right). Inset: AFM image (290 nm × 310 nm, W×H) showing a DONA aggregate. (B) SERS spectra showing measurements of individual DONA aggregates averaged over time as well as the total average calculated from all DONA aggregate measurements, compared to reference NR and SERS of CytC (vertical orange lines represent bands observed in the NR and SERS references and literature, summarized in Table SI1 and Table SI2; purple lines represent bands observed in our SERS reference measurements and not assigned in the literature). Each spectrum represents the time average of a measurement from a single DONA aggregate (blue zones highlight heme unit peaks, the red zone indicates peptide backbone peaks, and the purple zone shows spectral contributions from both). (C) SERS spectra showing different time points from a single DONA aggregate measurement, highlighting the shift of the Amide III band (blue zones highlight heme unit peaks, the red zone indicates peptide backbone peaks, and the purple zone shows spectral contributions from both).

Averaging the signals over the observation period revealed considerable variation among individual CytC spectra. Some spectra exhibited distinct CytC fingerprint peaks (Figure 4B, orange vertical lines), while others displayed a broader range of these peaks. This diversity aligns with expected results based on SERS reference spectra. Importantly, a clear oxidation state marker band at 1365 cm^–1^ was observed, most likely indicating a Fe^2+^ state, suggesting a reduction of the iron ion possibly due to electron transfer between the AuNPs and CytC. This peak provides a distinct contrast compared to the individual DONA measurements.

Similar to the single-DONA case (Figure 3C), we also observed a temporary spectral shift from approximately 1269 cm^–1^ to 1281 cm^–1^, lasting around 40 s before returning to 1269 cm^–1^ (Figure 4C). The Amide III band is a sensitive probe of protein secondary structure.^36,38^ The transient shift we observed, from approximately 1269 cm^–1^ to 1281 cm^–1^, falls within the characteristic frequency range (1265–1300 cm^–1^) associated with the α-helical secondary structure. The disappearance of peaks over time could indicate a reorientation of molecules within the hotspot, altering the vibrational modes that experience the greatest enhancement.

A deconvolution analysis was performed on the SERS reference spectrum (Figure SI1) and the average spectrum obtained from the DONA aggregates (Figure SI2). The deconvolution analysis revealed three main components in the Amide III band region. The peak at 1250 cm^–1^ is attributed to random coil conformations (36.8% in the SERS reference and 4.8% in the DONA aggregates). In contrast, the other two peaks, at 1269 and 1290 cm^–1^, are both characteristic of α-helical secondary structures (Table SI3; both together 63.1% in the SERS reference and 95.3% in the DONA aggregates). The different contributions could be related to the orientation of the bound CytC molecules within the hotspot. The α-helical regions might be positioned closer to the AuNPs in the DONAs, leading to a stronger enhancement of the corresponding vibrational modes compared to the reference spectrum.

Several factors could contribute to such dynamic behavior within our SERS measurements. Within the SERS hotspot, individual protein molecules might reorient or diffuse, altering their interaction with the electromagnetic field and contributing to peak shifts (Figure 5). Interactions between the protein and the metal surface can involve electron transfer processes, potentially affecting the protein’s electronic state and vibrational frequencies.^39^ Finally, even minor environmental fluctuations in temperature around the SERS hotspot can influence protein conformation and interactions with the metal surface, possibly contributing to the observed transient peak shift.^40,41^

**Figure 5:**
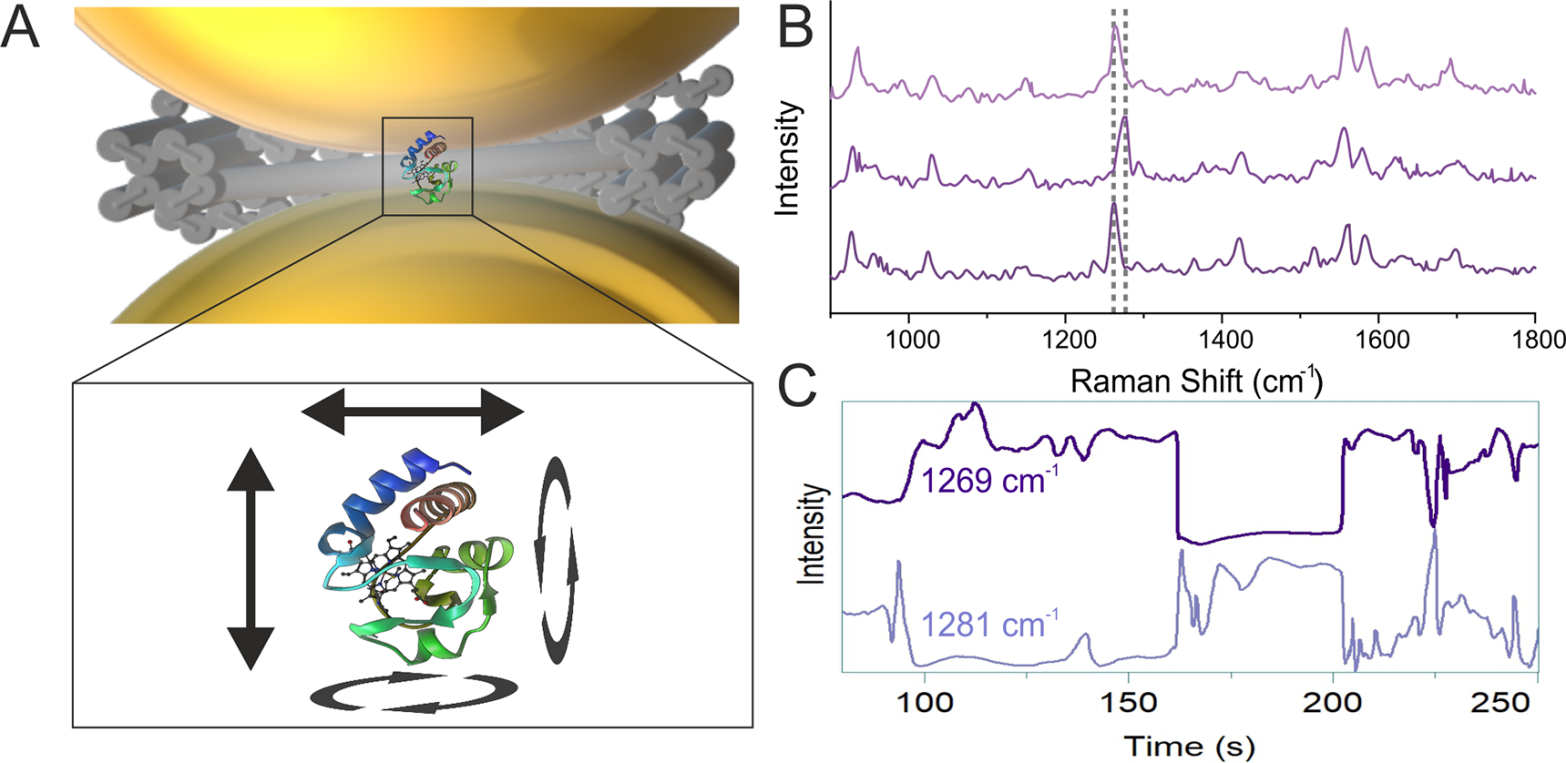
(A) Schematic of the CytC in the hotspot of the DONA and the possible reorientation or diffusion of the CytC inside the hotspot. (B) Spectra showing the shift from 1269 to 1281 cm^–1^ and back. (C) The time map visualizes the 1269 cm^–1^ peak (top, dark violet) and the 1281 cm^–1^ peak (bottom, light violet). A notable decrease in the 1269 cm^–1^ peak intensity around 160 s coincides with the emergence of the 1281 cm^–1^ peak. This pattern reverses around 200 s, indicating a transient shift between these two spectral positions. The structure of CytC is based on the crystal structure 2b4z deposited at the protein data bank (PDB): https://www.rcsb.org/structure/2b4z.

Additionally, in the DONA aggregate system, the observed peak shifts could arise from different CytC molecules within the aggregate experiencing varying degrees of enhancement. This variation stems from the likelihood of multiple CytC molecules being present in the hotspot, with their orientations and diffusion determining which molecule receives the greatest enhancement at a given moment. However, the transient decrease in the 1269 cm^–1^ peak intensity around 160 s, coinciding with the emergence of the 1281 cm^–1^ peak (Figure 5C), suggests that the spectra likely originate from a single CytC molecule. This is due to the matching intensities of the peak decrease and increase.

While the above-mentioned processes offer plausible explanations for the observed peak shifts, further analysis is needed to provide a definitive interpretation.^42^ Importantly, our measurements were conducted in a dry state, which limits the observation of protein folding dynamics. Future studies in solution environments, combined with the introduction of controlled stimuli like denaturants or temperature changes, could provide a more complete picture of protein conformational changes detectable through our technique.^43,44^

One significant advantage of our system, compared to UV resonance Raman techniques, is the reduced likelihood of protein denaturation. The persistent sharpness of the Amide III peaks throughout our experiment supports this claim, indicating that the protein structure remains largely intact. This advantage, along with the high sensitivity of SERS, highlights the potential of our technique for tracking subtle, real-time conformational changes in proteins within conditions closer to their native state.

In this study, we have demonstrated the potential of DONAs for single-molecule surface-enhanced Raman spectroscopy.^45^ Our results highlight the capability of this technique to detect subtle molecular interactions, opening new avenues for investigating intricate protein dynamics. Traditionally, observing nuanced shifts in peptide bond vibrational modes, particularly those associated with protein conformational changes, has been challenging due to limitations in ensemble measurements. Our system, with its enhanced sensitivity, facilitates the detailed observation of these protein dynamics in real-time. This could lead to valuable insights into protein function and interactions within complex environments.

A crucial advantage of our system is its ability to preserve sample integrity throughout the measurement period. This ensures that our observations reflect the protein’s native state, minimizing artifacts that could arise from degradation. Such preservation is vital for studying delicate proteins, leading to reliable and reproducible results.

## Supporting Information Available

Observed normal Raman and SERS bands and their assignments (Table SI1 and Table SI2); Materials and Methods (PDF)
