# Supplementary material for: From Bulk to Single Molecules: Surface-Enhanced Raman Scattering of Cytochrome C Using Plasmonic DNA Origami Nanoantennas
Source: Nano Lett. 2024 Jun 3;24(23):6916–23. doi: 10.1021/acs.nanolett.4c00834 (PMC11177308; doi:10.1021/acs.nanolett.4c00834)
Supplement: Supplementary file 1 — nl4c00834_si_001.pdf [file nl4c00834_si_001.pdf]

Supporting Information

From Bulk to Single Molecules - Surface-Enhanced Raman Scattering of Cytochrome C using Plasmonic DNA Origami Nanoantennas

Amr Mostafa,[a] Yuya Kanehira[a], Kosti Tapio[a,1], Ilko Bald[a]*

[a] Institute of Chemistry, University of Potsdam, Potsdam, 14469, Germany

*correspondence: ilko.bald@uni-potsdam.de

Table SI1. Observed normal Raman bands and band assignments.

**Regular Raman**					**Band assignment**
**514 nm[1]**		**532 nm[2]**		Our result	
Oxidized	Reduced	Oxidized (Low Spin)	Oxidized (High Spin)	532 nm	
		1128	1128	1126	ν_s(pyr half-ring)
1174		1169	1169	1169	ν_s(pyr half-ring)
1232		1240	1233	1230	δ(CH)
1314		1314	1316	1310	δ(CH)
1375	1360	1370	1371	1364,1378	ν_s(pyr half-ring)
1400		1403	1406	1394	ν(pyr quarter-ring)
1408					ν(pyr quarter-ring)
1501	1492	1499		1497	ν(CC)
1540				1540	ν(CC)
1568		1564		1556	ν_{as}(CC)
1587		1585	1568	1581	ν_{as}(CC)
1626					ν_{as}(CC)
1640		1637	1632	1633	ν_{as}(CC)

[1] Current address: Department of Neuroscience and Biomedical Engineering, Aalto University, Espoo, 02150, Finland

Table SI2. Observed SERS bands and band assignments.

SERS					Band assignment
**532 nm[2]**	**532 nm[3]**		Our result	Our result	
Oxidized	Oxidized	Reduced	532	561	
1131	1130	1130	1128	1132	ν_s(pyr half-ring)
1166	1172	1172	1171	1174	ν_s(pyr half-ring)
1232			1232	1230	δ(CH)
1315	1317	1316	1312	1314	δ(CH)
1379	1373	1363	1352,1374	1367	ν_s(pyr half-ring)
1401	1403	1400	1394	1397	ν(pyr quarter-ring)
1569	1569		1563		ν_{as}(CC)
1580	1585	1549	1546,1579	1544,1586	ν_{as}(CC)
1628	1638	1608		1608,1638	ν_{as}(CC)

Peak deconvolution

Peak deconvolution analysis was performed using the Peak Deconvolution app in Origin software. Initial peak positions were manually selected, followed by an automated initial fit. Peak centers were fixed, and full width at half maximum (FWHM) values were adjusted to a maximum value between 15 and 50 cm^{-1}. The app then iteratively refined the fit until convergence. Resulting peak areas were used to calculate the percentage contribution of each peak (Table SI3). This procedure was applied to both the SERS reference spectrum (Figure SI1) and the average spectrum of DONA aggregates (Figure SI2).

Table SI3. Deconvolution Analysis of the Amide III Band.

Raman shift (cm^{-1})	SERS ref. Area %	DONA agg. Area %	Amide III band
1250	36.8	4.8	Random coils
1269	14.3	54.9	Alpha-helix
1290	48.8	40.4	Alpha-helix

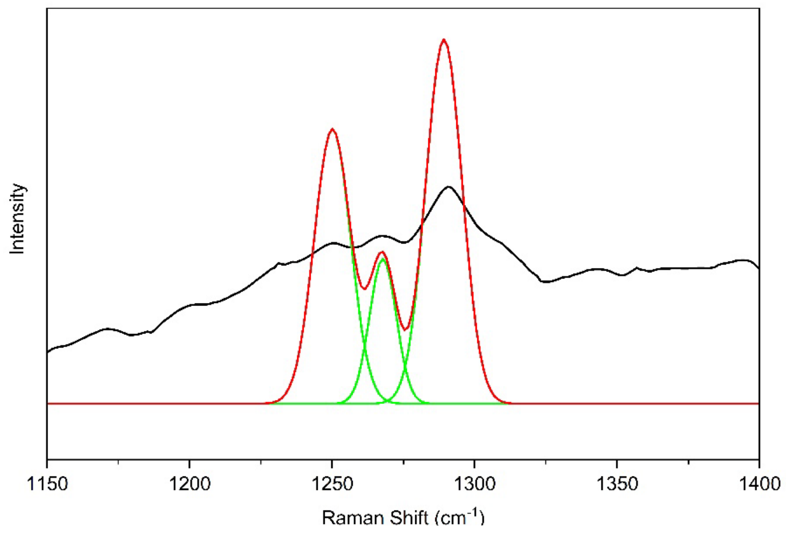

Figure SI1. Deconvolution analysis of the Amide III band. Black line: SERS reference spectrum. Red line: Sum of the deconvoluted peaks. Green lines: Individual deconvoluted peaks corresponding to 1250 cm^{-1} (random coil), 1269 cm^{-1} (alpha-helix), and 1290 cm^{-1} (alpha-helix).

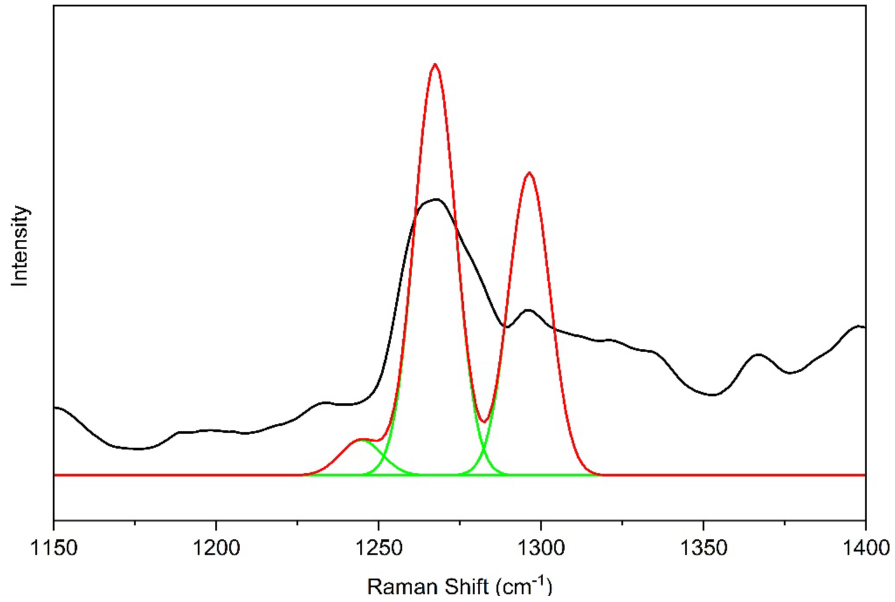

Figure SI2. Deconvolution analysis of the Amide III band. Black line: DONA aggregates average spectrum. Red line: Sum of the deconvoluted peaks. Green lines: Individual deconvoluted peaks corresponding to 1250 cm^{-1} (random coil), 1269 cm^{-1} (alpha-helix), and 1290 cm^{-1} (alpha-helix).

Materials & Methods

UV/Vis absorption

Cytochrome C (CytC) from bovine heart powder (purity ≥ 95%) was obtained from Sigma Aldrich and dissolved in ultrapure water to prepare a 100 µM stock solution. UV/Vis absorbance spectra were acquired using a NanoDrop 2000 Spectrophotometer (Thermo Scientific). A 2 µL aliquot of the CytC solution was analyzed using the instrument's predefined settings for protein measurements.

Bulk measurements

1. Normal Raman measurements

A small amount of powdered CytC was placed directly onto a clean silicon chip. The chip was secured onto a magnetic disc and analyzed using a HORIBA OmegaScope imaging instrument equipped with a LabRAM HR Evolution spectrometer. To prevent sample burning, laser power and integration time were optimized for each excitation wavelength as follows:

457 nm: 3.8 mW, 0.5 s

532 nm: 0.85 mW, 0.5 s

561 nm: 0.9 mW, 0.5 s

633 nm: 0.7 mW, 1 s

660 nm: 0.6 mW, 1 s

785 nm: 3.4 mW, 0.5 s

2. SERS measurements

A 400 µL aliquot of 60 nm gold nanoparticles (AuNPs), as obtained from the manufacturer, was centrifuged at 3500 g for 5 minutes at room temperature. The supernatant was carefully removed, and the pellet was resuspended in 25 µL of ultrapure water, resulting in a final AuNP concentration of approximately 0.3 nM. A 5 µL volume of 100 µM CytC solution was added to the AuNP solution, inducing rapid aggregation as evidenced by a visible color change. The mixture was drop-cast onto a clean silicon chip and air-dried for 1 hour.

The prepared sample chip was secured onto a magnetic disc and analyzed using the HORIBA OmegaScope with LabRAM HR Evolution spectrometer. Laser power and integration time were optimized to prevent sample burning, with the following settings used for each excitation wavelength:

532 nm: 0.085 mW, 0.5 s

561 nm: 4.5 mW, 0.5 s

633 nm: 0.1 mW, 1 s

660 nm: 0.6 mW, 0.1 s

785 nm: 2.1 mW, 0.5 s

DNA origami fork assembly

The DNA origami fork is assembled through a one-pot self-assembly process. This begins with combining 2.5 nM of the M13mp18 circular scaffold strand, which contains 7249 nucleotides, with 100 nM of 201 short oligonucleotides listed. These components are mixed in 1x TAE buffer, supplemented with 15 mM $MgCl_2$, and the total volume is brought up to 100 µL with ultrapure water.

The annealing process is carried out in a thermocycler. It starts with rapid heating to 80 °C, followed by a gradual cooling: first from 80 °C to 20 °C at a rate of 1 °C per 12 minutes, then from 20 °C to 16 °C at 1 °C per 6 minutes. Finally, the mixture is quickly cooled from 16 °C to 8 °C.

To remove excess staples, the mixture is processed through 100 kDa MWCO Amicon filters. This involves adding 100 µL of the DNA origami solution to the filters, followed by 400 µL of ultrapure water, and then centrifuging at 6000 g for 8 minutes at room temperature. The filtrate is discarded, and the filtering process is repeated twice more with 400 µL of ultrapure water each time.

The purified nanostructure solution is collected by inverting the filter into a new tube and centrifuging at 1000 g for 2 minutes at room temperature. This solution can be stored at 8 °C for up to two weeks.

Cytochrome C functionalization of DNA origami structures

Following DNA origami fork assembly and purification, a 5 µL aliquot of a 100 µM Cytochrome C (CytC) solution was added to 25 µL of the fork solution and incubated for 15 minutes at room temperature. This incubation step facilitates binding of CytC to the DNA origami structures. Subsequently, the mixture was purified twice using the same protocol employed for initial fork purification. This purification step removes unbound CytC, ensuring specific attachment to the DNA origami structures.

Gold nanoparticles (AuNP) coating

A modified version of the protocol by Liu et al[4]. was employed, involving a freezing step for the AuNP-DNA solution. The process begins with centrifuging 400 µL of 60 nm diameter AuNP solution, as provided by the manufacturer, at 3500 g for 5 minutes at room temperature. The supernatant is then removed using a pipette, and the resulting pellet is resuspended in 25 µL of ultrapure water, yielding a final AuNP concentration of approximately 0.3 nM.

Next, 1 µL of 100 mM Tris-(2-Carboxyethyl)phosphine (TCEP) solution is added to 4 µL of thiol-modified DNA (100 µM as supplied by the manufacturer). This mixture is incubated for 10 minutes at room temperature. Following incubation, the 5 µL mixture is added to the concentrated AuNP solution from step 2.1. The solution is then briefly vortexed for 5 seconds and frozen for a minimum of 2 hours at -20 °C.

After thawing the mixture at room temperature, it is centrifuged at 3500 g for 5 minutes at room temperature to remove excess coating DNA. The supernatant is then discarded, and the pellet is resuspended in 10 µL of water.

It should be noted that there are two different coating strands, one for each of the AuNPs on the nanofork. The steps for coating are the same, except for the sequence of the DNA coating strand.

DONA assembly

For the assembly of DONAs, a series of carefully calibrated steps is followed. Initially, the coated AuNPs solution is added to the nanofork solution in a molar concentration ratio of 1.5:1. Following this, $MgCl_2$ is introduced to reach a final concentration of 4 mM, utilizing a 50 mM $MgCl_2$ stock solution, and the total volume is adjusted to 20 µL with ultrapure water. The hybridization of the DONAs is then facilitated through a controlled temperature gradient in a thermocycler. This involves a rapid initial heating to 40 °C, followed by a gradual cooling from 40 °C to 20 °C at a rate of 1 °C every 10 minutes, and concluding with a swift cooling from 20 °C to 8 °C. This temperature control is crucial for the proper folding and assembly of the DONAs.

Gel electrophoresis

Agarose gel electrophoresis is utilized to remove unbound nanoparticles from the DONA solution. The process begins with the preparation of a 1% agarose gel, where 0.8 g of agarose is dissolved in 80 mL of 1x TAE buffer, enhanced with 5 mM $MgCl_2$. To ready the DONA solution for electrophoresis, 2.25 µL of loading buffer, containing 30% glycerol and 13 mM MgCl2, is added to 18 µL of the DONA solution. This adjustment increases the $MgCl_2$ concentration to a final value of 5 mM. The loading buffer also ensures the sample stays within the gel pocket during the electrophoresis process. The gel is then run for 60 minutes at 70 V, in an ice water bath, using 1x TAE buffer with 5 mM $MgCl_2$ as the running buffer. After running the gel, the band of interest is extracted by cutting it from the gel and placing it on a paraffin plastic film-wrapped microscopy slide. The solution is then squeezed out using a second wrapped slide and collected into a 500 µL tube with a pipette. This step is crucial for isolating the DONAs, ensuring they are free from unbound nanoparticles.

Colocalization of AFM and Raman measurements.

Initially, a silicon chip is plasma-treated for 10 minutes. Following this, a mixture of 10 µL of the purified DONA solution and 10 µL of 100 mM $MgCl_2$ is incubated on the chip for 3 hours. After incubation, the chip is washed twice with a 1:1 mixture of ethanol and water and then blow-dried using compressed air. The chip is then secured onto a magnetic disc for insertion into the imaging instrument (HORIBA OmegaScope with a LabRAM HR evolution).

Before proceeding with the measurements, the position of the Raman excitation laser is adjusted to align directly above the AFM probe tip. This ensures precise colocalization of the Raman and AFM measurements. For the AFM measurements, the tapping mode (AC mode) is employed using ACCESS-NC-A tips, which have a resonance frequency of 300 kHz and a spring constant of 45 N/m. The AC mode is advantageous as it automatically controls all parameters, except for the scan rate, which is manually set to 1 Hz.

After completing the AFM imaging, the AFM tip is retracted to prevent it from obstructing the path of the Raman laser. This retraction is accomplished using a pre-programmed macro function titled “Probe away” (AIST/LabSpec). This setup guarantees that for any selected point in the AFM image, the laser positioning will perfectly correspond with no offset compared to the AFM data, thus enabling accurate and synchronized AFM and Raman analyses.

The concluding step in this process is the careful determination of the laser wavelength, power, and accumulation time, tailored to the unique characteristics of each sample. For single DONA measurements, the settings include a 660 nm laser wavelength, a power level of 3.5 mW, and an integration time of 0.1 seconds over a total duration of 300 seconds. In contrast, when measuring aggregates, these parameters are slightly modified. While the laser wavelength is retained at 660 nm, the power is decreased to 1.4 mW, and the integration time is kept constant at 0.1 seconds for 300 seconds.

References

(1) Barth, A. Infrared Spectroscopy of Proteins. *Biochim. Biophys. Acta - Bioenerg.* **2007**, *1767* (9), 1073–1101. https://doi.org/10.1016/J.BBABIO.2007.06.004.

(2) Yeo, B. S.; Mädler, S.; Schmid, T.; Zhang, and W.; Zenobi, R. Tip-Enhanced Raman Spectroscopy Can See More: The Case of Cytochrome C. *J. Phys. Chem. C* **2008**. https://doi.org/10.1021/jp709799m.

(3) Kühler, P.; Roller, E. M.; Schreiber, R.; Liedl, T.; Lohmüller, T.; Feldmann, J. Plasmonic DNA-Origami Nanoantennas for Surface-Enhanced Raman Spectroscopy. *Nano Lett.* **2014**, *14* (5), 2914–2919. https://doi.org/10.1021/NL5009635/SUPPL_FILE/NL5009635_SI_001.PDF.

(4) Liu, B.; Liu, J. Freezing-Driven DNA Adsorption on Gold Nanoparticles: Tolerating Extremely Low Salt Concentration but Requiring High DNA Concentration. *Langmuir* **2019**, *35* (19), 6476–6482. https://doi.org/10.1021/ACS.LANGMUIR.9B00746/SUPPL_FILE/LA9B00746_SI_001.PDF.
